# Diseases of the Dental Pulp

**Published:** 1883-04

**Authors:** Samuel A. White

**Affiliations:** Savannah, Ga.


### ARTICLE IV.

### *Treatment of Diseases of the Dental Pulp.*

BY DR. SAMUEL A. WHITE, SAVANNAH, GA.

Among the many operations that the practicing dentist is called upon to perform, there are none probably of a more important character than the restoration to health and usefulness of teeth that have their lining membrane exposed, or their vitality gone.

The dental pulp in its various conditions of exposure, together with the treatment necessary to relieve our patients of continued and occasionally almost unendurable suffering, should at all times receive our very best attention ; and in offering these few observations upon the general treatment of exposed pulps, and devitalized teeth, I do not claim any particular originality, but would rather offer some practical evidence and the mode of treatment that I have found most successful.

In what I would term a simple or partial exposure of the pulp, without inflammation, the treatment is generally simple, and nearly always successful; where there has been no pain, or the exposure has been caused by accident in excavating, I proceed to remove all decayed dentine from around the edges of the cavity, cautiously approaching the exposed spot, and prepare it at once for a permanent filling. A drop or two of blood showing, instead of being unfavorable, is in many cases a positive advantage, but every indication of it should cease before the filling is commenced. This can be readily acccomplished with spirits of camphor. In this condition, however, as well as in all other exposures of the pulp, there is one consideration of the utmost importance, and that is, that no particle of detached dentine be left to press upon the pulp to produce irritation, or failure would be inevitable. If the cavity is plainly visible and everything prepared for filling, it should be wiped out with alcohol and creosote of equal parts, to be followed with creosote alone, or dilute carbolic acid, then carefully dry and place two or three thicknesses of No. 60 gold, previously cut to fit the floor of the cavity, immediately over exposure, and proceed to fill, first using rather large pellets of soft foil about No. 5, pressed well against the marginal walls, and gradually bringing them over the heavy foil, which should at first be held in position either by an assistant or an instrument held in the left hand. In every case the filling should be finished with cohesive gold, as it is almost impossible either to dove-tail the pellets or introduce them as an ordinary soft foil filling. If the cavity is inaccessible, or particularly deep, or if amalgam is to be used, proceed as above described, only substituting six or eight thicknesses of paper, moistened with creosote, for the heavy foil, and fill with oxy-phosphate zinc, this to be capped with the harder material.

The next condition of exposed pulp, is where pain has been experienced for a greater or less time, produced either by slight thermal changes, suction or pressure. If at the time of examination there is the slightest pain or uncomfortable symptoms, they should be allayed. Chloroform and creosote of equal parts I have found most to be relied upon, except in excessive pain when creosote alone should be used. In the former condition I proceed at once to fill temporarily. Prepare by excavating the marginal walls of the cavity as for permanent filling, leaving that portion over exposure almost untouched, place a small pellet of cotton saturated with creosote next the pulp, removing excess with bibulous paper, and fill with oxy-phosphate zinc. This should remain at least a week or ten days, when, if no trouble is experienced, the whole should be removed and several thicknesses of bibulous paper substituted for the cotton, and a permanent filling introduced. In the other condition, where acute inflammation has been of long duration, I regret to say that the chances of success are not very favorable, although with a little care and perseverance, many teeth of this character can be restored to health and made useful for years. Creosote should be applied once or twice a day until all uneasy symptoms entirely disappear, and the tooth remain in a perfectly comfortable condition for several days. Treatment should then be proceeded with as just described. Care should always be exercised in mixing the oxy-phosphate of zinc somewhat thinner than for permanent work, and using as little pressure as possible in its introduction. Failure here brings us to the consideration of the next condition of pulp exposure, when it becomes necessary to destroy its vitality. In cases of this kind the treatment and subsequent success is very much more assured, although among regular patients I am happy to say seldom resorted to. After reducing all inflammation, by one or more applications of creosote, or either of the essential oils, the cavity should be prepared to retain a filling material. The smallest speck of cotton, not larger than a mustard seed, merely touched in arsenious paste, should be placed directly upon the exposed point of pulp, and filled over with thin oxy-phosphate of zinc. The most scrupulous care should be observed that no particle of the paste touch the gum or soft parts of the mouth. This should remain in the cavity about twenty-four hours. Upon removing it, care should be used not to bruise or break up the pulp but simply insert a pellet of cotton moistened with creosote and dismiss the patient for forty-eight hours, at which time there will be little or no difficulty in removing the pulp almost entire, by first cutting away the approach to pulp-chamber and then using a barbed broach.

Now comes the most important step, and careless manipulation will only induce failure. In reaming out the nerve-canal, neither air, debris, or point of instrument should, under any circumstances, be allowed to penetrate beyond the apex. I believe that nine-tenths of unsuccessful cases can be traced to want of care in this particular. With the excellent nerve-drills now obtainable, which clear themselves while cutting, there is no excuse for either stopping up the canal or forcing debris through the foramen. As it is all important that these delicate instruments should not break while using them, it would be well to test each in the following manner: Clamp the cutting or bur end between two pieces of soft pine in a vise, and with the fingers turn the handle, using rather more force than would be attempted within the tooth.

In all nerve cavity cases, except those having an outlet through the alveolus and gum, and I may also except the six front teeth, of which I shall speak hereafter, it is necessary, in their treatment, to fill the fangs in such a manner that the entire filling can be readily removed in the event of trouble. This should be provided for in every case.

The material I have employed the past six or eight years for this purpose is floss silk. The following quotation is from a paper I read at our last State meeting in reference to use of this material:

"In the treatment of dead teeth that have no fistula, I find lightly waxed floss silk, with which to temporarily fill fangs, the most useful and satisfactory of anything I have ever tried. After the fangs are properly prepared and wiped out with creosote, take a piece of floss about two inches long, placing an end in the cavity, then with a perfectly smooth nerve broach catch the end of the silk and gently press it up to the apex; withdrawing the broach a little, press it up again; the silk will invariably go with it. Repeat this until the fang or fangs are completely filled, leaving an end in the cavity by which the whole can be readily removed whenever necessary. It is always best to use a separate piece for each root. In lower bicuspids and molars, when the root can be opened at all, the silk will be found to be particularly suitable, the flatness not preventing the almost perfect adaptation of the silk. Possibly its chief merit is that no air is ever pumped up with it, and the ease with which it can be removed. I believe it can also be used successfully as a permanent filling for roots, certainly when trouble is anticipated at some future day."

In the sixth front teeth I think better results are obtained by the use of wood, both as a temporary and permanent filling. The ease with which it can be accurately fitted, both as regards length and size of root, making it particularly suitable. As a temporary filling the end should project from the cavity just sufficient to be grasped by a pair of delicately pointed forceps. After inserting the wood well up to the end of the root, cotton should be packed around it and the whole hermetically sealed with gutta-percha stopping. This should remain at least ten days or two weeks, before the permanent filling is inserted. If there is the slightest indication, however, of periostitis, the gums adjacent should be freely painted with iodine, to which one-fourth part tinct. aconite root has been added. Care should be used to prevent the iodine extending further than desired. If inflammation continues, remove the filling and commence a more conservative treatment. By that I mean a more gradual sealing of the cavity. This can be done by passing well up into the root a medium size piece of twine, around which cotton should be firmly packed, the first piece dipped in creosote, the whole being capped with gutta-percha stopping; the twine is then withdrawn, leaving a small open canal which will gradually become closed by the flattening of the filling. The gums should be painted once a day until all unfavorable symptoms disappear.

As a permanent filling for roots that are round or nearly so, well seasoned hickory I believe to possess advantages superior to anything else. We frequently find that the nerve canal near the apex is so exceedingly small that it is almost impossible to force any filling material up to the end of the root, at least with certainty, and again so large that we are in danger of forcing the filling through the foramen. In both of these conditions wood, I believe, will be found to work with certainty. After being satisfied as regards its perfect adaptation to the root to be filled, by actual trial, pass the edge of a knife lightly around the wood about one-fourth of an inch from the end to be inserted, bend it over at this point without breaking, and then insert; a few slight taps with a mallet will send it home, and a slight twist between the fingers will break it off at the place marked by the knife, and the root is permanently filled even to the apex. Either gold, gutta-percha or oxy-phosphate of zinc may follow, although I prefer the last two in the order named, which gives a solid foundation for the gold.

We come now to another and more troublesome condition of devitalized teeth. Those from which a discharge has been going on possibly for years, where there is no fistulous opening or ever has been, and while they may have given no trouble whatever, the slightest attempt at closing up the cavity is followed by serious disturbance, which if persisted in, results in periosteal inflammation and alveolar abscess, and while in a few cases this might be somewhat excusable, very much doub tits advisability in any case. My practice has been to "make haste slowly," first being careful to remove all foreign substance from the roots, and cleaning them out as perfectly as possible without closing them up, then thoroughly inject tepid water to be followed with alcohol. Creosote should be introduced on a piece of cotton which must be packed very lightly as far up as possible, this to be changed every day, packing the cotton more firmly at each sitting. As soon as the tooth will submit to it, fill, leaving an open canal as already described. The gums should be painted with the iodine preparation every day. If, after a tooth has been permanently filled, active periosteal inflammation should take place, depletion by freely scarifying the gums; painting the scarified surface with iodine will sometimes give relief. If, however, this should fail, and the tooth is a valuable one, then trephining the alveolus will have to be resorted to. This can best be accomplished with a sharp spear-shaped drill; the only pain experienced is in passing through the soft parts.

In the next and last condition of which I shall speak, where there is a fistulous opening and a clear passage can be established through the cavity, the treatment is simple. Force either creosote alone, or combined with an equal quantity of iodine through the cavity until it is plainly seen coming from the fistula, and fill, permanently, which can be done with perfect safety. An easy method to accomplish this, is to take a pellet of cotton saturated with the preparation and place it well into the cavity, all excess next the wall taken up, and a piece of gutta-percha stopping, warmed and flattened placed over the cavity. This should be sufficiently soft to adhere to every part of the tooth with which it comes in contact. A stout bur or drill should previously be wrapped tightly with cotton, just to comfortably fit the cavity. Place this in the center of the gutta-percha, and gently but firmly press it into the cavity, carrying the gutta-percha and cotton before it. If the passage is clear, this will be found to work with perfect satisfaction and in the most positive manner.—*Southern Dental Journal.*

